# Sexual dimorphism in an adaptive radiation: Does intersexual niche differentiation result in ecological character displacement?

**DOI:** 10.1002/ece3.8137

**Published:** 2021-10-02

**Authors:** Benjamin D. Wasiljew, Jobst Pfaender, Benjamin Wipfler, Mariam Gabelaia, Ilham Vemandra Utama, Letha Louisiana Wantania, Fabian Herder

**Affiliations:** ^1^ Zoologisches Forschungsmuseum Alexander Koenig Bonn Germany; ^2^ Naturkundemuseum Potsdam Potsdam Germany; ^3^ Division of Zoology Ichthyology Laboratory Research Center for Biology Indonesian Institute of Sciences (LIPI) Cibinong Indonesia; ^4^ Faculty of Fisheries and Marine Science Sam Ratulangi University Manado Indonesia

**Keywords:** 3D geometric morphometrics, adaptive radiation, ecological character displacement, intersexual niche differentiation, sexual dimorphism, *Telmatherina*

## Abstract

Evolutionary radiations are one plausible explanation for the rich biodiversity on Earth. Adaptive radiations are the most studied form of evolutionary radiations, and ecological opportunity has been identified as one factor permitting them. Competition among individuals is supposedly highest in populations of conspecifics. Divergent modes of resource use might minimize trophic overlap, and thus intersexual competition, resulting in ecological character displacement between sexes. However, the role of intersexual differentiation in speciation processes is insufficiently studied. The few studies available suggest that intersexual niche differentiation exists in adaptive radiations, but their role within the radiation, and the extent of differentiation within the organism itself, remains largely unexplored. Here, we test the hypothesis that multiple morphological structures are affected by intersexual niche differentiation in “roundfin” *Telmatherina*, the first case where intersexual niche differentiation was demonstrated in an adaptive fish radiation. We show that sexes of two of the three morphospecies differ in several structural components of the head, all of these are likely adaptive. Sexual dimorphism is linked to the respective morphospecies‐specific ecology and affects several axes of variation. Trait variation translates into different feeding modes, processing types, and habitat usages that add to interspecific variation in all three morphospecies. Intrasexual selection, that is, male–male competition, may contribute to variation in some of the traits, but appears unlikely in internal structures, which are invisible to other individuals. We conclude that intersexual variation adds to the adaptive diversity of roundfins and might play a key role in minimizing intersexual competition in emerging radiations.

## Graphical abstract

Here, we test the hypothesis that multiple morphological structures are affected by intersexual niche differentiation in “roundfin” *Telmatherina*, the first case where intersexual niche differentiation was demonstrated in an adaptive fish radiation. We show that sexes of two of the three morphospecies differ in several structural components of the head, all of these are likely adaptive. Trait variation translates into different feeding modes, processing types, and habitat usages that add to interspecific variation in all three morphospecies. We conclude that intersexual differentiation adds to the adaptive spectrum of roundfins, and might play a key role in minimizing intraspecific competition in emerging radiations.

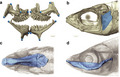

## 1 INTRODUCTION

The concept of evolutionary radiation, evolutionary divergence of a single lineage into a variety of different adaptive forms, is one plausible explanation for the rich biodiversity on Earth (Naciri & Linder, 2020; Nosil, 2012; Simões et al., 2016). Some of the best‐studied examples of evolutionary radiations are adaptive radiations (Gavrilets & Losos, 2009; Losos, 2010; Naciri & Linder, 2020; Simões et al., 2016), which are driven by the evolution of ecological divergence and the accumulation of reproductive isolation (Martin & Richards, 2019; Rundle & Nosil, 2005; Schluter, 2009; Schluter & Conte, 2009). The evolution of morphological adaptations that enable alternative modes of ecological resource use may facilitate coexistence of closely related species in different ecological niches (Losos, 2010; Martin & Richards, 2019; Nosil, 2012; Schluter, 2000; Yoder et al., 2010).

Ecological competition is expectedly highest in populations of conspecifics (McGee et al., 2020). Divergent selection has been shown to play a key role in interspecific adaptive processes (Rundle & Nosil, 2005; Schluter, 2009) and might even be an important factor for divergence between sexes (De Lisle, 2019; De Lisle & Rowe, 2017; Roy et al., 2013). Divergent modes of resource use minimize intersexual competition for limited trophic resources, resulting in ecological character displacement between males and females (De Lisle, 2019; De Lisle & Rowe, 2017; Roy et al., 2013). Although the role of intersexual variation in speciation processes has gained little attention so far (De Lisle, 2019; De Lisle & Rowe, 2017; Ronco et al., 2019), the few studies available suggest that intersexual niche differentiation is present in adaptive radiations (De Lisle & Rowe, 2017; Pfaender et al., 2011; Ronco et al., 2019). It has been demonstrated, for instance, in *Anolis* lizards, salamanders, and sticklebacks (Butler, 2007; De Lisle & Rowe, 2017; McGee & Wainwright, 2013). Whether intersexual niche differentiation rather retards or promotes adaptive radiations is still an ongoing discussion (Bolnick & Doebeli, 2003; Butler, 2007; De Lisle & Rowe, 2015, 2017), but recent studies have shown that ecological speciation and ecological character displacement can occur simultaneously (De Lisle & Rowe, 2015, 2017). However, the actual role of intersexual niche differentiation in species flock formation remains largely unexplored (De Lisle, 2019; De Lisle & Rowe, 2017; Pfaender et al., 2011; Ronco et al., 2019).

Sexual dimorphism is widespread in adaptive radiations (Herler et al., 2010; McGee & Wainwright, 2013), most commonly as sexual size dimorphism or sexual color dimorphism (Herler et al., 2010; Tsuboi et al., 2012). It can either be induced by sexual selection, by intrinsic differences between males and females, or by intersexual competition (De Lisle, 2019; Hedrick & Temeles, 1989; Herler et al., 2010), whereby these drivers may interact in many cases of sexual dimorphism (Bolnick & Doebeli, 2003; Temeles et al., 2000). Sexual selection mechanisms provide plausible explanations for many of the spectacular cases, but cannot account for intersexual phenotypic variation in general (De Lisle, 2019; Hedrick & Temeles, 1989; Tsuboi et al., 2012). This is especially true for ecologically relevant traits and internal structures (Bolnick & Doebeli, 2003; De Lisle, 2019; Ronco et al., 2019). In these cases, it seems rather plausible that intersexual competition for ecological resources is a main cause for the development of sexual dimorphism (Bolnick & Doebeli, 2003; Ronco et al., 2019). Examples of sexual dimorphism in ecologically relevant traits include stick insects, hummingbirds, *Anolis* lizards, salamanders, cichlids, and sticklebacks (Albert et al., 2008; Butler, 2007; De Lisle & Rowe, 2017; Herler et al., 2010; Hulsey et al., 2015; McGee & Wainwright, 2013; Ronco et al., 2019; Roy et al., 2013; Temeles & Kress, 2003).

The cranial region of fishes contains key traits for food acquisition, ranging from size and shape of the skull to variation in gill rakers, oral and pharyngeal jaws, opercle, and the buccal cavity (Burress et al., 2016, 2018; Carroll et al., 2004; Hellig et al., 2010; Ronco et al., 2019; Rösch et al., 2013; Wilson, et al., 2013; Wilson, et al., 2013). These structural components have been identified as ecologically relevant and likely adaptive traits toward feeding mode, habitat, and prey items in fish radiations (Burress et al., 2016, 2018; Carlig et al., 2018; Carroll et al., 2004; Cook, 1996; Hellig et al., 2010; Hulsey et al., 2006; Wilson, Colombo, et al., 2013; Wilson et al., 2015; Wilson, Furrer, et al., 2013). For instance, previous studies on cichlids, sticklebacks, and catfishes have shown that the shape and size of the opercle can be highly correlated with lifestyle and feeding mode (Stange et al., 2016; Wilson, Colombo, et al., 2013; Wilson et al., 2015; Wilson, Furrer, et al., 2013). The opercle pump helps to create a pressure gradient at the mouth opening and a current across the gills supporting the respiratory system (Kimmel et al., 2012; Wilson, Colombo, et al., 2013; Wilson et al., 2015; Wilson, Furrer, et al., 2013). A large opercle is beneficial for suction feeding performance and respiration performance of benthic living fishes; these typically live at stationary bottom waters and are usually less mobile (Kimmel et al., 2012; Wilson, Colombo, et al., 2013; Wilson et al., 2015; Wilson, Furrer, et al., 2013). The shape and dentition of the pharyngeal jaw has been shown to be strongly adapted to different prey types in several cichlids and sailfin silversides (Burress, 2016; Burress et al., 2016, 2018; Hellig et al., 2010; Pfaender et al., 2010). Species feeding on hard‐shelled prey tend to have a sturdy pharyngeal jaw with enlarged bones and teeth adapted to crushing (Burress, 2016; Burress et al., 2016, 2018; Grubich, 2003; Hulsey et al., 2006; Wainwright, 2005), while fish‐feeding species typically have elongated and slender pharyngeal jawbones with few, large teeth adapted for grasping (Burress, 2016; Burress et al., 2016, 2018; Hellig et al., 2010; Pfaender et al., 2010). Previous studies on nototheniids, centrarchids, and cottid fishes have shown that buccal cavity size can differ according to prey size and mobility (Carlig et al., 2018; Carroll et al., 2004; Cook, 1996). The buccal cavity is crucial for generating a suction pressure that draws prey items through the mouth opening, and its diameter limits the maximum prey size (Carlig et al., 2018; Carroll et al., 2004; Cook, 1996; Mihalitsis & Bellwood, 2017). A large buccal cavity is advantageous for suction feeders feeding on elusive prey because it can produce a higher pressure gradient, which is effective over distance. Although these adaptive patterns have been identified in several fish radiations, they have mainly been investigated on an interspecific level. However, in radiations where intersexual niche differentiation is documented, these patterns might also be detectable on an intersexual level.

**FIGURE 1:**
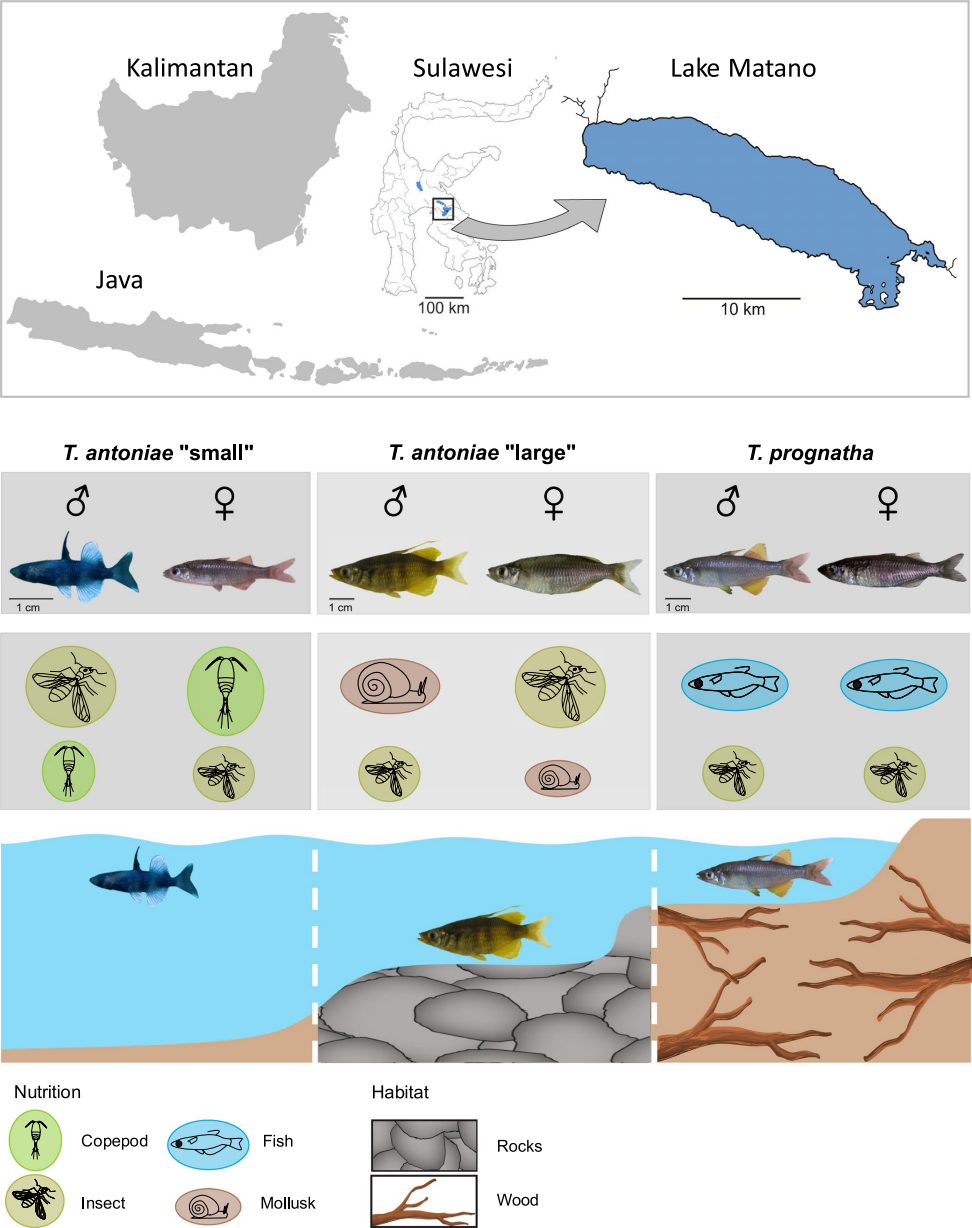
Indonesia, Sulawesi, and Lake Matano with the three endemic roundfin morphospecies *T. antoniae* “small”, *T. antoniae* “large”, and *T. prognatha*. Adult, reproducing males and females are pictured with key aspects of their ecological differentiation. Nutrition size corresponds to the respective relevance in diet composition. Map by T. von Rintelen, modified (with permission). This figure has been designed using resources from Freepik.com

“Roundfin” *Telmatherina* are a small monophyletic group within the radiation of sailfin silversides and are endemic to ancient Lake Matano located in the central highlands of Sulawesi (Figure 1) (Herder, Nolte, et al., 2006; Herder et al., 2006, 2008; von Rintelen et al.,  2012). Roundfins consist of three morphospecies, *Telmatherina antoniae* “small”, *Telmatherina antoniae* “large”, and *Telmatherina prognatha* (Kottelat, 1991). According to distance‐based divergence estimates and molecular clock analyses, the initial divergence of these morphospecies occurred around 1 My (Stelbrink et al., 2014). However, AFLP genotyping revealed that reproductive isolation among the three morphospecies is substantial but incomplete (Herder, Nolte, et al., 2006; Herder Pfaender & Schliewen, 2008; Herder & Schliewen, 2010; Herder, Schwarzer, et al., 2006). Roundfins show significant interspecific differences in body and head shape, and a pronounced sexual dimorphism (Pfaender et al., 2011; Wasiljew et al., 2020). All three morphospecies occupy different microhabitats and specific trophic niches (Figure 1) (Herder et al., 2008; Herder & Schliewen, 2010; Pfaender et al., 2011): *T. antoniae* “small” is a pelagic, predominantly planktivorous suction feeder; *T. antoniae* “large” is a predominantly benthic, mollusk‐eating suction feeder; and *T. prognatha* is a semipelagic, mainly fish eating ram feeder. Roundfin *Telmatherina* were also the first case where intersexual niche differentiation was demonstrated in an adaptive fish radiation (Pfaender et al., 2011). Two of the three morphospecies (*T. antoniae* “large” and “small”) show intersexual niche differentiation in trophic ecology, while *T. prognatha* does not (Pfaender et al., 2011). Male *T. antoniae* “small” take a significantly higher portion of terrestrial insects compared with females, which are more specialized on copepods. Male *T. antoniae* “large” consume a higher percentage of mollusks than females, which take a higher amount of terrestrial insects (Figure 1) (Pfaender et al., 2011). Other dietary components are rather negligible in these two morphospecies (Pfaender et al., 2011). However, these intersexual niche differences have not been linked to morphological structures relevant for prey processing and habitat usage so far.

Here, we test the hypothesis that multiple morphological structures are affected by intersexual niche differentiation in roundfin *Telmatherina*. Variation in three structural components—the opercle, the pharyngeal jaw, and the buccal cavity—which are directly linked to prey capture, prey processing, and habitat usage in fishes, was studied with µ‐CT Imaging. This technique enables one to investigate particularly small‐scaled variation of internal structures with great detail and precision without damaging the samples (Adams et al., 2004; Kaliontzopoulou, 2011; Wake, 2012; Wasiljew et al., 2020). Interspecific and intersexual variation was analyzed using different three‐dimensional morphometric approaches ranging from classical measurements of distances to landmark‐free geometric, morphometric analyses. We hypothesized that the opercle, the pharyngeal jaw, and the buccal cavity are adaptive in roundfins, with specific adaptations to resource use in the respective species and sexes. Further, we predicted that the degree of intersexual variation should coincide with the degree of intersexual niche differentiation in each morphospecies.

## 2 MATERIALS AND METHODS

### 2.1 Material and µ‐CT imaging

The present study was based on formalin‐fixated roundfin *Telmatherina* specimens that were available from collection material. These were obtained in the dry season of 2002 from three locations around Lake Matano's shoreline, using gill nets (Figure 1). Since the specimens used in this study were gathered from museum collection material, no living animals were sampled, killed, harmed, or treated in any other way for this paper.

The skulls of 13 specimens of each morphospecies *T. antoniae* “small”, *T. antoniae* “large”, and *T. prognatha* were used for 3D µ‐CT analyses. µ‐CT scanning was performed with Skyscan 1272 and Skyscan 1173 scanners (Bruker). All specimens were scanned in 70% ethanol. Five male and five female specimens per species were stained with 0.3% phosphotungstic acid (PTA) in advance. Five male and five female specimens per species were scanned without any prior staining. The resolution ranged between 11 µm and 23 µm depending on the size of the specimen. Selected rotation steps varied between 0.2, 0.3, and 0.4 degrees over 180°. The chosen voltage ranged between 60 kV and 100 kV and the current between 80 µA and 166 µA. Detailed scanner settings for each individual can be viewed in Dryad. The projections were reconstructed with NRecon ver. 1.7.1.0 (Bruker). Data size was then reduced with the software Dataviewer ver. 1.5.2.4 by Bruker and ImageJ ver. 1.51f by NIH (Schindelin et al., 2015). Segmentation and volume rendering of the resulting 3D models were accomplished with the software packages Amira ver. 6.5.0 by Thermo Fisher Scientific (Stalling et al., 2005) and VG Studio 3.2 by Volume Graphics. Surface rendering was performed with the software packages Checkpoint ver. 17.04.21 (Stratovan Corporation) and Amira ver. 6.5.0 by Thermo Fisher Scientific (Stalling et al., 2005). Final plates were arranged with Adobe Photoshop CS6 and Adobe Illustrator CS6.

### 2.2 Classical and geometric morphometrics

To identify variation in the opercle bone and the pharyngeal jaw, linear morphometric measurements and geometric morphometric analyses were conducted based on surface‐rendered 3D models created by the software Checkpoint ver. 17.04.21 (Stratovan Corporation) out of µ‐CT tiff image stacks. The following traits of the cranial skeleton were quantified by linear measurements: skull length, left opercle height, left opercle length, left opercle circumference, left opercle surface area, lower right pharyngeal jaw length, lower right pharyngeal jaw width, lower right pharyngeal jaw height, and lower right pharyngeal jaw circumference. The number of teeth on the right lower pharyngeal jaw was counted. All measurements were carried out with the software Checkpoint.

**FIGURE 2:**
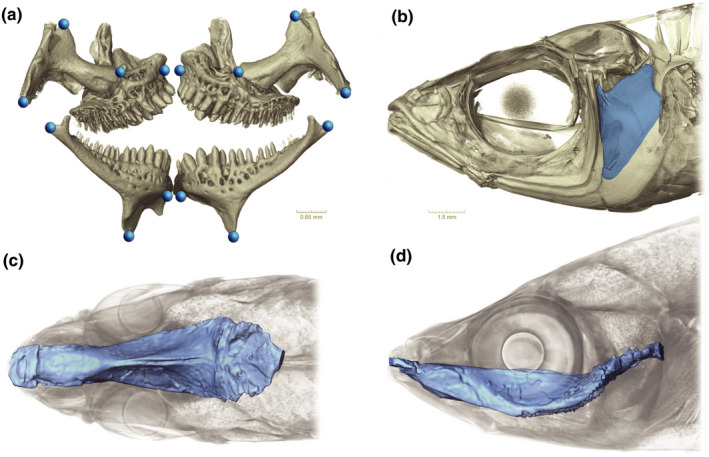
(a–d) Three analyzed structural components of roundfin *Telmatherina*. (a) Surface‐rendered 3D model of the pharyngeal jaw of *T. prognatha*. Locations of 14 homologous landmarks placed on the pharyngeal jaws of µ‐computed tomography‐scanned roundfin specimens. (b) Surface‐rendered 3D model of the head of *T. prognatha*. Location of the opercle (colored in blue) of roundfin specimens (*n* = 10). The outline was used for quantifying the circumference and the shape of the opercle. (c, d) Surface‐rendered 3D model of the buccal cavity (in blue) is shown within a volume render (in gray) of a previously stained *T. antoniae* “small”. (c) Dorsal view; (d) lateral view. The surface render was used for the quantification of size and volume of the buccal cavity

In order to test for interspecific and intersexual shape differences, 14 landmarks were placed at homologous points on the pharyngeal jaws of the 30 unstained µ‐CT scanned specimens (Figure 2a). The outline shape and circumference of the left opercle (Figure 2b) and the pharyngeal jaw were analyzed with 80 semilandmarks. Patches were used to measure the surface area of the opercle in order to quantify its overall size between species and sexes.

### 2.3 Buccal cavity measurements

All classical morphometric measurements and geometric morphometric analyses of the buccal cavity were based on surface‐rendered 3D models created by Amira ver. 6.5.0 by Thermo Fisher Scientific (Stalling et al., 2005) out of µ‐CT tiff image stacks. In order to quantify interspecific and intersexual variation in buccal cavity size and shape, the 30 stained specimens were used for creating volume‐rendered models of the cranial region with the software Amira. Surface‐rendered models of the buccal cavity were created with the help of the semiautomatic segmentation tool of Amira (Figure 2c,d). Background artifacts were removed by applying the “remove islands” and “fill holes” options of Amira on the segmented 3D model. The length, width, height, and volume of the buccal cavity were measured for every prior‐stained specimen. Two female specimens of *T. prognatha* were removed from the analysis because they showed deformations of the buccal cavity due to a slightly opened mouth.

Due to the rather featureless structure of the buccal cavity, surface scans were used instead of landmarks to describe its shape. A landmark‐free shape analysis of the buccal cavity was performed by the Generalized Procrustes Surface Analysis (GPSA) software package in Java executable (version 20200722 provided by B. J. Pomidor upon personal request) (Pomidor et al., 2016; Slice, 2013). Surface renders were superimposed through iterative closest point (ICP) algorithm. After the superimposition, the homologous point coordinates were subjected to dimension reduction and the principal axis scores were calculated for the further analysis (Pomidor et al., 2016).

### 2.4 Statistical analyses

Bivariate linear models were performed for the absolute measurements of the opercle, pharyngeal jaw, and buccal cavity in order to control for size in each trait. The absolute measurements of the opercle and the buccal cavity were regressed with skull length. The absolute measurements of the pharyngeal jaw were regressed with overall pharyngeal jaw width. The absolute number of pharyngeal teeth was regressed with pharyngeal jaw circumference. In order to test for significant differences between species and sexes, the resulting residuals of each bivariate linear model were used to perform one‐way ANOVAs with Tukey's pairwise tests in the software PAST ver. 3.22 (Hammer et al., 2001). Species and sexes were tested simultaneously, resulting in six groups per model and trait.

The shape data of the opercle and pharyngeal jaw were analyzed with Procrustes superimposition followed by an elliptic Fourier analysis (EFA) respectively principal component analysis (PCA) and thin‐plate spline interpolation performed in the software PAST ver. 3.22 (Hammer et al., 2001) and R ver. 3.5.1 (Ihaka & Gentleman, 1996). The resulting scores of the PCA and EFA in the three first axes were used to perform a MANOVA and a Tukey's pairwise test with PAST in order to test for significant differences between species and in the dataset. In order to test for intersexual shape differences, this procedure was repeated for each species dataset individually, instead of a pooled‐species dataset. This was done to avoid the disproportionate influence of more variable species on the principal axes over the less variable ones.

## 3 RESULTS

The skulls of *T. antoniae* “large” (mean: 18.18 mm) and *T. prognatha* (mean: 19.49 mm) were significantly larger than the skulls of *T. antoniae* “small” (mean: 10.58 mm; *Q* = 20.83; *p* = <.01), meeting the documented size ranges by Herder et al. (2008). Intersexual differences in skull length were only detectable in *T. antoniae* “large” (*Q* = 5.91; *p* = <.01). Male *T. antoniae* “large” (mean: 19.62 mm) had significantly larger skulls than females (mean: 16.72 mm).

Size variances for different structures and parameters were unequally portioned between species and sexes. All the absolute and the majority of relative size measurements were more divergent interspecifically than intersexually. The exceptions were the relative height and surface area of the opercle, the relative circumference of the pharyngeal jaw, and the relative height of the buccal cavity. In these parameters, intersexual variance exceeded the variance among species. Detailed size variance ratios are provided in Dryad. All analyzed structures differed significantly in at least one size parameter between morphospecies. Interspecific size variation was most distinct for the pharyngeal jaw, while intersexual size variation was most distinct for the opercle. Shape variation was most distinct for the buccal cavity among species and sexes. Intersexual size differences were significant in both *T. antoniae* morphospecies but not in *T. prognatha*. Intersexual differences in shape were present in all three morphospecies.

### 3.1 Opercle

**FIGURE 3:**
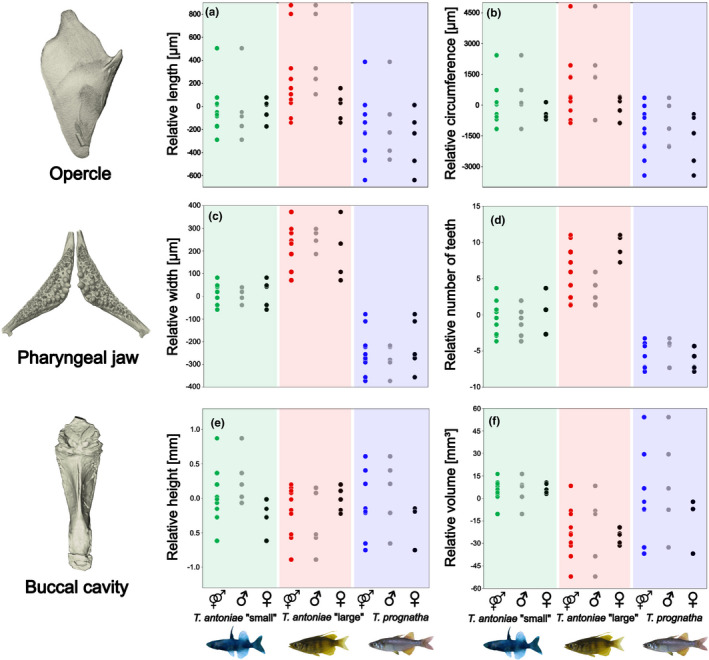
(a–f) Interspecific and intersexual variation in relative (a) opercle length, (b) opercle circumference, (c) pharyngeal jaw width, (d) number of pharyngeal teeth, (e) buccal cavity height, and (f) buccal cavity volume of roundfin *Telmatherina* (species *n* = 10; sex *n* = 5). Dots visualize single individuals. Morphospecies and sexes are color‐coded: *T. antoniae* “small” combined—green; *T. antoniae* “large” combined—red; *T. prognatha* combined—blue; male—gray; and female—black. Two female specimens of *T. prognatha* were removed from the buccal cavity analysis because they showed deformations due to a slightly opened mouth

**FIGURE 4:**
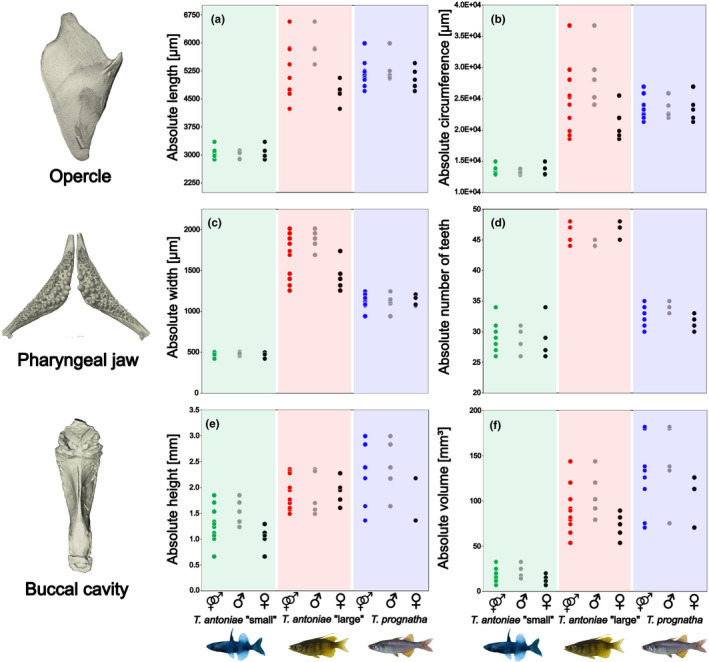
(a–f) Interspecific and intersexual variation in absolute (a) opercle length, (b) opercle circumference, (c) pharyngeal jaw width, (d) number of pharyngeal teeth, (e) buccal cavity height, and (f) buccal cavity volume of roundfin *Telmatherina* (species *n* = 10; sex *n* = 5). Dots visualize single individuals. Morphospecies and sexes are color‐coded: *T. antoniae* “small” combined—green; *T. antoniae* “large” combined—red; *T. prognatha* combined—blue; male—gray; and female—black. Two female specimens of *T. prognatha* were removed from the buccal cavity analysis because they showed deformations due to a slightly opened mouth

**FIGURE 5:**
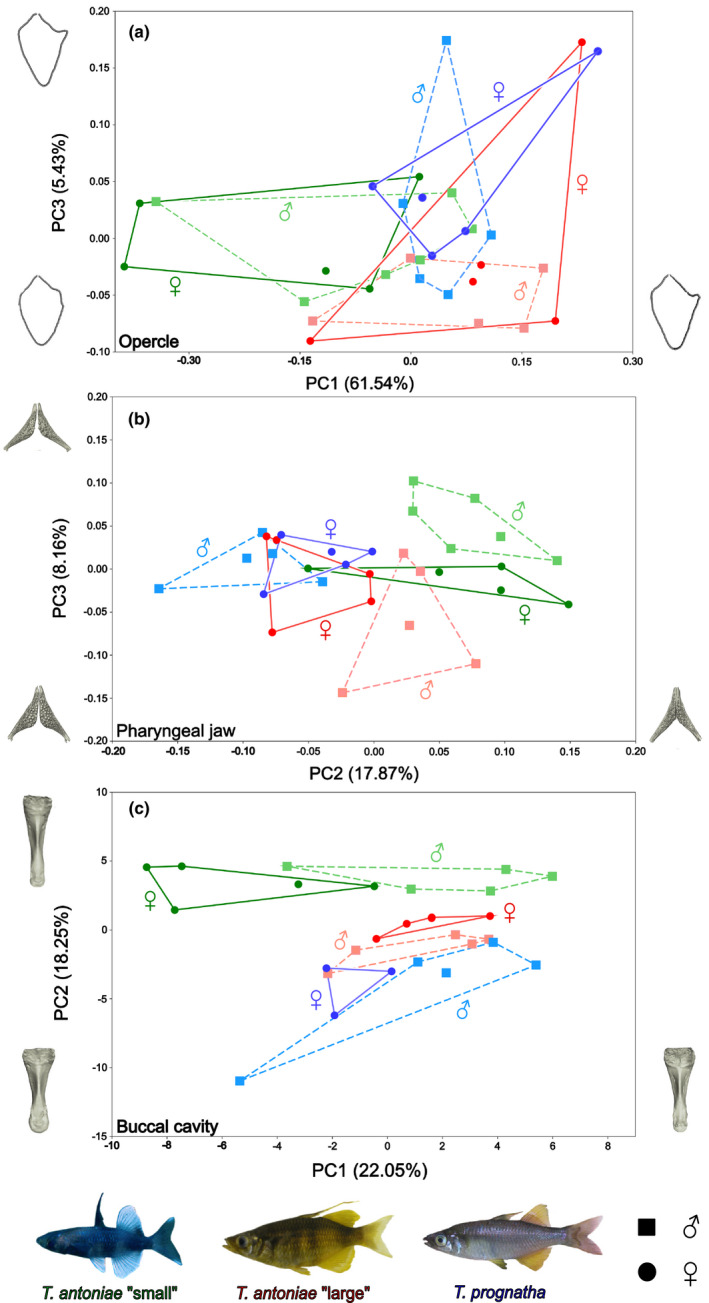
(a–c) Interspecific and intersexual variation in shape of the (a) opercle, (b) pharyngeal jaw, and (c) buccal cavity of roundfin *Telmatherina* with visualizations of the mean shapes for each species (species *n* = 10; sex *n* = 5). (a) Elliptic Fourier analysis plot of the opercle semilandmark data set with point clusters of species and sexes. (b) Principal component analysis plot of the pharyngeal jaw landmark data set with point clusters of species and sexes. (c) Principal component analysis plot of the buccal cavity shapes data set with point clusters of species and sexes. Morphospecies are color‐coded, sexes are symbol‐coded (*T. antoniae* “small”—green; *T. antoniae* “large”—red; *T. prognatha* —blue; male—square; female—dot). Two female specimens of *T. prognatha* were removed from the buccal cavity analysis because they showed deformations due to a slightly opened mouth

**FIGURE 6:**
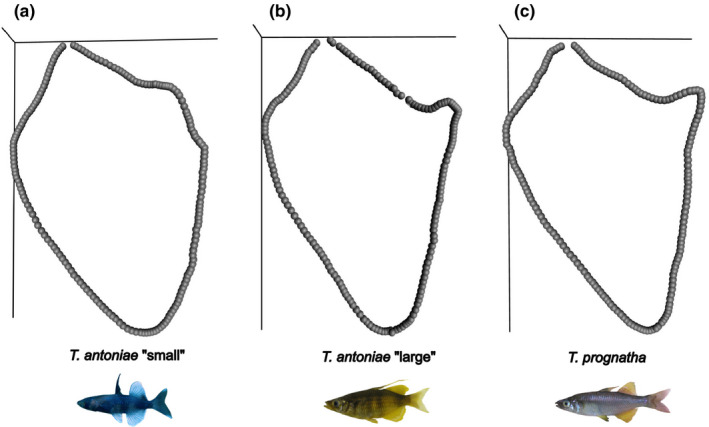
(a–c) Mean outline shape of the opercle of (a) *T. antoniae* “small”; (b) *T. antoniae* “large”; and (c) *T. prognatha*

Relative opercle size differed substantially between species. Following the predictions based on the species‐specific niches, it was highest in *T. antoniae* “large”, followed by *T. antoniae* “small”, and *T. prognatha* (Figure 3a,b; *Q* = 5.19; *p* = <.05). Absolute opercle size was significantly lower in *T. antoniae* “small” in comparison with *T. antoniae* “large” and *T. prognatha* (Figure 4a,b; *Q* = 17.62; *p* = <.01). No significant differences in opercle size were identified between the latter two morphospecies. The shown values of relative length and circumference also reflect the patterns for the remaining not visualized parameters. In contrast, the morphospaces of the opercle shape EFA showed a large overlap of all three morphospecies without any significant differences in shape within the first three axes (Figure 5a). However, the opercle outline of *T. antoniae* “small” could be distinguished from the other morphospecies by its round shape (*F* = 4.62; *p* = <.05). *Telmatherina antoniae* “large” and *T. prognatha* shared a rather triangular‐shaped opercle (Figure 6).

This discrepancy between size and shape was also detected among sexes. Male and female *T. antoniae* “small” and *T. prognatha* did not differ in relative or absolute opercle size (Figures 3a,b and 4a,b; *Q* = 1.57; *p* = >.1). Consistent with the higher percentage of mollusks in their diet (Figure 1), males of *T. antoniae* “large” had significantly higher relative and absolute values than females (Figures 3a,b and 4a,b; *Q* = 7.64; *p* = <.05). In contrast, intersexual variation in shape was not substantial (*F* = 0.66; *p* = >.05). The morphospaces of the EFA comprising the sexes distinctly overlapped within all morphospecies (Figure 5a).

### 3.2 Pharyngeal jaw

**FIGURE 7:**
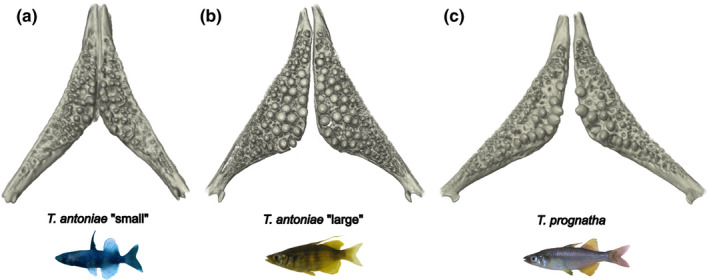
(a–c) Pharyngeal jaws in dorsal view of (a) *T. antoniae* “small”; (b) *T. antoniae* “large”; and (c) *T. prognatha*

Relative pharyngeal jaw size differed significantly in height, width, and relative number of teeth (*Q* = 19.25; *p* = <.05) but not in length or circumference between species (Figure 3c,d). The shown values of relative width and number of teeth were the most discriminative parameters. In line with the proportion of mollusks in its diet (Figure 1), *T. antoniae* “large” was characterized by a sturdy pharyngeal jaw with a high relative number of teeth. As predicted for a piscivorous predator (Figure 1), *T. prognatha* had a gracile pharyngeal jaw with a low relative number of teeth. Planktivorous *T. antoniae* “small” was intermediate in size, shape, and relative number of teeth (Figures 3c,d and 7). Absolute pharyngeal jaw size was lowest in *T. antoniae* “small,” highest in *T. antoniae* “large,” and intermediate in *T. prognatha* (Figure 4c,d; *Q* = 23.30; *p* = <.01). Pharyngeal jaw shape differed substantially between morphospecies and was distinctly separated by the morphospaces of the PCA in the first three axes (Figure 5b; *F* = 10.63; *p* = <.05).

Intersexual variation was absent in relative pharyngeal jaw size in all morphospecies (Figure 3c) but present in the relative number of teeth within *T. antoniae* “large” (Figure 3d; *Q* = 7.86; *p* = <.01). Sexes of *T. antoniae* “small” and *T. prognatha* did not differ in absolute pharyngeal jaw size, while males of *T. antoniae* “large” had significantly larger pharyngeal jaws than females (Figure 4c; *Q* = 6.22; *p* = <.05), consistent with the higher proportion of mollusks in their diet (Figure 1). Sexual dimorphism in pharyngeal jaw shape was present within all three morphospecies. The morphospaces of male and female specimens were distinctly separated (Figure 5b), but shape variation was only significant in *T. antoniae* “large” (*F* = 15.45; *p* = <.05).

### 3.3 Buccal cavity

**FIGURE 8:**
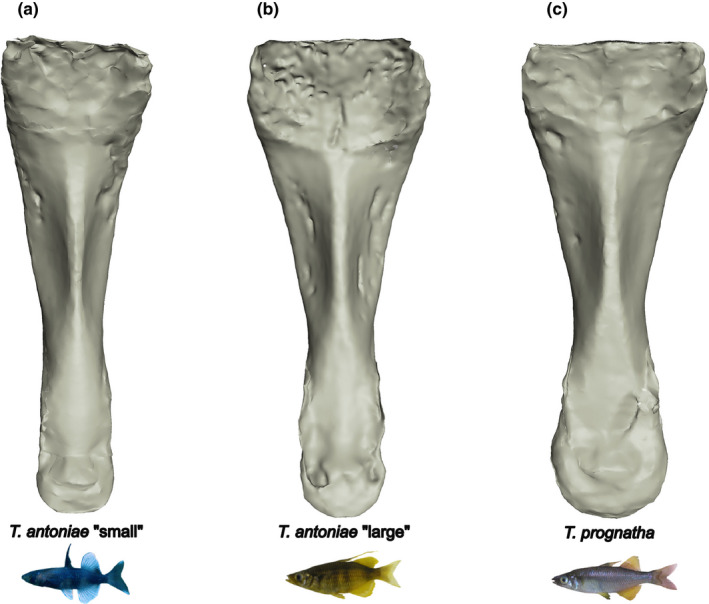
(a–c) Mean buccal cavities in dorsal view of (a) *T. antoniae* “small”; (b) *T. antoniae* “large”; and (c) *T. prognatha*

Morphospecies differed in relative buccal cavity volume, but not in any of the relative linear measurements (Figure 3e,f). In line with the predictions according to feeding mode and diet composition (Figure 1), *T. antoniae* “small” had the largest, *T. antoniae* “large” the smallest, and *T. prognatha* an intermediate relative buccal cavity volume (Figure 3f; *Q* = 4.66; *p* = <.05). The visualized values of relative height and volume were the most discriminative parameters. Absolute buccal cavity size was lowest in *T. antoniae* “small”, highest in *T. prognatha,* and intermediate in *T. antoniae* “large” (Figure 4e,f; *Q* = 22.25; *p* = <.01). Buccal cavity shape differed substantially between morphospecies (Figure 8), while the morphospaces of the PCA were significantly separated in the first three axes (Figure 5c; *F* = 14.54; *p* = <.01).

Intersexual variation in relative and absolute buccal cavity size was significant within *T. antoniae* “small” and *T. antoniae* “large”. Compared with females, relative buccal cavity height was higher in male *T. antoniae* “small”, consistent with the higher percentage of copepods in their diet (Figure 1), but lower in male *T. antoniae* “large” (Figure 3e; *Q* = 3.98; *p* = <.05), in line with the higher proportion of mollusks in their diet (Figure 1). Absolute size was higher in male *T. antoniae* “small” and *T. antoniae* “large” than in females (Figure 4e,f; *Q* = 5.01; *p* = <.05). Sexual dimorphism in shape was most distinct for the buccal cavity. The morphospaces of the PCA comprising the sexes were distinctly separated within all morphospecies (Figure 5c), but shape variation was only significant in *T. antoniae* “small” and “large” (*F* = 13.95; *p* = <.05).

## 4 DISCUSSION

### 4.1 Ecologically relevant traits in roundfins

The present study revealed significant differences between roundfin sailfin silverside morphospecies in three structural components of the head region. All of these are ecologically relevant and are considered adaptive in other fish radiations (Burress et al., 2016, 2018; Carlig et al., 2018; Carroll et al., 2004; Cook, 1996; Hellig et al., 2010; Hulsey et al., 2006; Wilson, Colombo, et al., 2013; Wilson et al., 2015; Wilson, Furrer, et al., 2013).

As expected for predominantly benthic, mollusk‐eating, suction‐feeding fishes (Burress et al., 2016, 2018; Cook, 1996; Muller et al., 1982; Wainwright, 2005; Wilson, Colombo, et al., 2013; Wilson, Furrer, et al., 2013), *T. antoniae* “large” shows a large, triangularly shaped opercle (Figures 3a,b, 5a, and 6), a wide, sturdy‐shaped pharyngeal jaw with a high relative number of teeth (Figures 3c,d, 5b and 7), and a small buccal cavity (Figures 3e,f and 5c). The semipelagic, mainly fish eating ram feeder *T. prognatha* is characterized by having a small, triangularly shaped opercle (Figures 3a,b, 5a and 6), a narrow, gracile pharyngeal jaw with a low relative number of teeth (Figures 3c,d, 5b and 7), and an intermediate‐sized buccal cavity (Figures 3e,f and 4c). These findings are characteristic for pelagic, ram feeding predators (Burress et al., 2016, 2018; Carroll et al., 2004; Hellig et al., 2010; Wilson, Colombo, et al., 2013; Wilson et al., 2015; Wilson, Furrer, et al., 2013). *Telmatherina antoniae* “small” shows characteristic patterns for a suction‐feeding fish with a pelagic lifestyle and a planktivorous diet (Cook, 1996; Hulsey et al., 2006; Pfaender et al., 2010; Pfaender et al., 2011; Wilson et al., 2015) with its intermediate‐sized and roundly shaped opercle (Figures 3a,b, 5a and 6), gracile pharyngeal jaw (Figures 3c,d, 5b and 7), and relatively large buccal cavity compared with *T. antoniae* “large” and *T. prognatha* (Figures 3e,f and 5c).

### 4.2 Intersexual trait variation meets predictions derived from ecology

Sexual dimorphism may affect feeding ecology, and intersexual niche differentiation might minimize intraspecific competition in radiations (De Lisle, 2019; Pfaender et al., 2011; Roy et al., 2013). Intersexual variation in ecological adaptive traits has been reported in various animal groups (Butler, 2007; Cooper et al., 2011; De Lisle & Rowe, 2017; Maan & Seehausen, 2011), but most studies focus on size or color dimorphism, which can be induced by sexual selection rather than ecological divergent selection (De Lisle, 2019; Hedrick & Temeles, 1989; Herler et al., 2010; Tsuboi et al., 2012). In order to identify ecological‐based intersexual divergent selection, it is of major interest to investigate ecologically relevant traits in well‐documented cases of intersexual niche differentiation (Bolnick & Doebeli, 2003; De Lisle, 2019; De Lisle & Rowe, 2017; Ronco et al., 2019).

Among Lake Matano's roundfins, sexual dimorphism in ecologically relevant traits is most pronounced in *T. antoniae* “large”. Males have relatively larger opercles (Figure 3a,b), a lower number of teeth, more sturdy pharyngeal jaws (Figures 3d and 5b), and flatter buccal cavities than females (Figures 3e,f and 5c). This coincides with sex‐specific trophic profiles: Males feed more pronounced on mollusks than females, while females feed to a higher extent on insects than males (Pfaender et al., 2011). The conspicuously sturdy pharyngeal jaws of males with a low number of large teeth (Figures 3c,d and 5b) are considered advantageous for dealing with hard‐shelled prey (Burress, 2016; Burress et al., 2016, 2018; Hellig et al., 2010), while a large buccal cavity, as it occurs in female *T. antoniae* “large”, is considered advantageous for catching elusive prey via a suction feeding mode (Carroll et al., 2004). The relatively larger opercle of male *T. antoniae* “large” is discussed to be an adaptation to benthic suction feeding (Wilson, Colombo, et al., 2013; Wilson et al., 2015; Wilson, Furrer, et al., 2013), enhancing active ventilation of the gills, which is considered advantageous for reduced motility in static waters (Kimmel et al., 2008; Wilson, Colombo, et al., 2013; Wilson, Furrer, et al., 2013). This fits the more benthic lifestyle of male *T. antoniae* “large”, compared with female conspecifics (Pfaender et al., 2011), and matches findings of an adaptive sexual dimorphism in sticklebacks, which likewise differ in the use of both benthic versus limnetic habitats and opercle traits (Albert et al., 2008; McGee & Wainwright, 2013).

In contrast to *T. antoniae* “large”, male and female *T. antoniae* “small” differ in size and shape of the buccal cavity (Figures 3e and 5c), but not in opercle or pharyngeal jaw traits (Figures 3a‐d and 5a,b). Males have significantly higher and deeper buccal cavities than females (Figures 3e and 5c). Again, these findings match the sex‐specific trophic niches: Male *T. antoniae* “small” feed to a larger extent on insects than females, which are predominantly zooplanktivores (Pfaender et al., 2011). Both prey types differ significantly in size (Pfaender et al., 2011; Wainwright & Bellwood, 2002), which matches the differences detected in buccal cavity size and shape (Figures 3e and 5c). The diameter of the buccal cavity generally limits both the maximum prey size and the suction pressure (Carlig et al., 2018; Carroll et al., 2004; Cook, 1996; Mihalitsis & Bellwood, 2017). Therefore, large buccal cavities are advantageous for fish species feeding on large prey items (Carroll et al., 2004; Mihalitsis & Bellwood, 2017). Male *T. antoniae* “small” might benefit from a larger buccal cavity compared with females since they take a higher percentage of insects, which are substantially larger than zooplankton (Pavlov & Kasumyan, 2002; Pfaender et al., 2010, 2011; Wainwright & Bellwood, 2002). The studies of Herler et al. (2010) and Ronco et al. (2019) reported sexual dimorphism in the buccal cavity of mouth‐brooding cichlid fishes. However, they linked intersexual variation to parental care but not to different trophic niches. The present case is hence, to the best of our knowledge, the first study documenting sexual dimorphism in buccal cavity size and shape in a non‐mouth‐brooding fish radiation. This finding might support the ecological relevance of the buccal cavity in fish radiations. The absence of intersexual variation in opercle and pharyngeal jaw traits (Figures 3a‐d and 5a,b) may be explained by the generally similar requirements for taking insects and zooplankton (Pavlov & Kasumyan, 2002; Pfaender et al., 2010; Wainwright & Bellwood, 2002): Both prey types occur in the pelagic zone and share a similar texture (Pfaender et al., 2010, 2011; Wainwright & Bellwood, 2002). Thus, this trophic niche partitioning most likely does not affect intersexual variation in the opercle and pharyngeal jaw, which are linked to habitat usage and prey processing (Burress, 2016; Burress et al., 2016, 2018; Hellig et al., 2010; Kimmel et al., 2008; Wilson, Colombo, et al., 2013; Wilson et al., 2015; Wilson, Furrer, et al., 2013).

Conspicuously, the present study did not reveal indications for morphological differentiation among male and female *T. prognatha* (Figures 3 and 5). However, morphology also meets predictions derived from trophic ecology in this species (Pfaender et al., 2011). The absence of intersexual variation appears plausible since both sexes share similar trophic and habitat niches (Pfaender et al., 2011).

### 4.3 Does intersexual niche differentiation result in ecological character displacement?

The degree of sexual dimorphism detected here largely meets predictions derived from niche segregation in male and female roundfins (Figures 1, 3, and 5). It ranges from the absence of differences in ecologically relevant traits in *T. prognatha*, the species lacking intersexual niche differentiation, to *T. antoniae* “large”, where both intersexual trophic niches and trait segregation are most pronounced (Pfaender et al., 2011).

Sexual dimorphism can follow ecological‐based divergent selection or sexual selection (Hedrick & Temeles, 1989; Herler et al., 2010). Sexual selection can either affect display for potential mates (intersexual selection) or competitive advantages over other males (intrasexual selection) (Hedrick & Temeles, 1989; Herler et al., 2010; Tsuboi et al., 2012). Alternatively, intersexual variation can evolve by ecological selection pressure acting differentially on both sexes and thus favoring dimorphic niches (De Lisle, 2019; Hedrick & Temeles, 1989; Herler et al., 2010). An ecological cause for intersexual variation appears more plausible, if it occurs in traits likely affecting resource exploitation (Bolnick & Doebeli, 2003). The present analyses suggest that all three analyzed structures are likely ecologically adaptive in roundfins. Likewise, sexual dimorphism in the internal structures pharyngeal jaw and buccal cavity has probably evolved under ecological selection pressure, since these structures are of relevance for food acquisition (buccal cavity) and processing (pharyngeal jaw). In contrast to the majority of morphological traits analyzed in roundfins so far (Herder et al., 2008; Pfaender et al., 2011), both are nonvisible and thus unlikely to serve in signaling for potential mates or competitive males (Bolnick & Doebeli, 2003; Ronco et al., 2019). Nevertheless, we cannot exclude the possibility that sexual selection affects these structures in a nonvisual way, that is, through other signaling pathways or through allometric effects of sexual size dimorphism. However, as patterns of size and shape variation in the opercle, pharyngeal jaw, and buccal cavity detected in sticklebacks, cichlids, and other fish radiations (Albert et al., 2008; Burress, 2016; Burress et al., 2016, 2018; McGee & Wainwright, 2013) are also present in roundfins, it seems plausible that the identified variation is predominantly a result of ecological‐based divergent selection.

Intersexual ecological character displacement in both *antoniae* morphospecies might minimize trophic and habitat overlap (De Lisle, 2019; De Lisle & Rowe, 2017). For instance, the intersexual variation in the buccal cavity of *T. antoniae* “small” (Figures 3e and 5c) corresponds to different diet compositions between males and females (Carroll et al., 2004; Cook, 1996; Mihalitsis & Bellwood, 2017). Sexual dimorphism in all three investigated structures within *T. antoniae* ”large” (Figures 3 and 5) affects both habitat and diet composition between sexes (Burress, 2016; Burress et al., 2016, 2018; Hellig et al., 2010; Stange et al., 2016). Consequently, these morphological adaptations might reduce intersexual competition for ecological resources when resources are limited (De Lisle, 2019; De Lisle & Rowe, 2017), as in the case of this ultraoligotrophic lake (Herder & Schliewen, 2010; von Rintelen et al.,  2012). The absence of intersexual variation in *T. prognatha* (Figures 3 and 5), the only roundfin species without any reported intersexual niche differentiation (Pfaender et al., 2011), further supports this theory.

## 5 CONCLUSIONS

We demonstrate that roundfin morphospecies and sexes differ significantly in multiple ecologically relevant traits affecting prey capture, prey processing, and habitat use. As interspecific and intersexual variations meet patterns of niche differentiation reported in roundfins (Herder et al., 2008; Pfaender et al., 2011), these differentiations are likely adaptations to different ecological niches. Since the analyzed structural components are ecologically relevant and, in the case of the pharyngeal jaw and the buccal cavity, are invisible for other individuals, it seems unlikely that sexual selection is responsible for the intersexual variation documented here (Bolnick & Doebeli, 2003; De Lisle, 2019; De Lisle & Rowe, 2017; Ronco et al., 2019). It rather appears plausible that ecological‐based intersexual divergent selection is the main driver for the revealed intersexual variation in roundfins. The intersexual ecological character displacement in *T. antoniae* “small” and *T. antoniae* “large” likely minimizes trophic and habitat overlap and thus intersexual competition for ecological resources. Intersexual morphological differentiation adds to the adaptive diversity of roundfin *Telmatherina* and might play a key role in minimizing intersexual competition in emerging radiations. Further research is also needed on other systems to deepen our knowledge of the role of intersexual niche differentiation in speciation processes.

## CONFLICT OF INTEREST

The authors have no conflict of interest to declare.

## AUTHOR CONTRIBUTIONS

**Benjamin D. Wasiljew:** Conceptualization (equal); data curation (lead); formal analysis (equal); funding acquisition (lead); investigation (equal); methodology (equal); project administration (equal); software (equal); visualization (equal); writing‐original draft (lead); writing‐review & editing (lead). **Jobst Pfaender:** Conceptualization (equal); formal analysis (equal); supervision (supporting); writing‐original draft (supporting); writing‐review & editing (supporting). **Benjamin Wipfler:** Methodology (equal); software (equal); supervision (supporting); visualization (equal); writing‐original draft (supporting); writing‐review & editing (supporting). **Mariam Gabelaia:** Formal analysis (equal); methodology (equal); software (equal); writing‐original draft (supporting); writing‐review & editing (supporting). **llham Vemandra Utama:** Writing‐original draft (supporting); writing‐review & editing (supporting). **Letha Louisiana Wantania:** Writing‐original draft (supporting); writing‐review & editing (supporting). **Fabian Herder:** Conceptualization (equal); funding acquisition (supporting); investigation (equal); project administration (equal); resources (lead); supervision (lead); writing‐original draft (supporting); writing‐review & editing (supporting).

## Data Availability

Sampling locations, detailed scanner settings, absolute measurements, variance ratios, and landmark coordinates are provided at Dryad digital repository (https://doi.org/10.5061/dryad.0gb5mkm1x). All µ‐CT scans are deposited at our institute and can be requested by contacting the third author. If required, µ‐CT data can be uploaded at MorphoBank.
